# Analysis of Cytotoxic Granules and Constitutively Produced Extracellular Vesicles from Large Granular Lymphocytic Leukemia Cell Lines

**DOI:** 10.3390/cells13161310

**Published:** 2024-08-06

**Authors:** Lara Ploeger, Patrick Kaleja, Andreas Tholey, Marcus Lettau, Ottmar Janssen

**Affiliations:** 1 Molecular Immunology—Institute for Immunology, University Hospital Schleswig-Holstein, Campus Kiel, 24105 Kiel, Germany; 2 Systematic Proteomics & Bioanalytics—Institute for Experimental Medicine, University of Kiel, 24105 Kiel, Germany; 3 Stem Cell Transplantation and Immunotherapy—Internal Medicine II, University Hospital Schleswig-Holstein, Campus Kiel, 24105 Kiel, Germany

**Keywords:** T cells, NK cells, large granular lymphocyte leukemia (LGLL), cytotoxic granules, cytotoxic effector proteins, lysosome-related effector vesicles (LREV), extracellular vesicles (EV), exosomes, proteomics profiling

## Abstract

Background: Large granular lymphocyte leukemias (LGLLs) are rare lymphoproliferative malignancies caused by clonal expansion of granular lymphocytes. T-cell LGLL and natural killer (NK) cell LGLL are defined based on their cellular origin. Their clinical manifestation and pathophysiology vary depending on the subtype and include, e.g., neutropenia, anemia, recurrent infections, and autoimmunity. A limited number of available patient-derived cell lines are considered valuable tools to study the biology of these malignancies. They differ in the expression of lineage-specific surface markers, but generally contain cytotoxic effector molecules in characteristic granules. Methods: We investigated the presence and release of lysosome-associated effector proteins in patient-derived LGLL cell lines by flow and imaging cytometry, by Western blotting and by bottom–up proteomics profiling. Results: The tested cell lines did not express FasL (CD178), but did express CD26/DPP4^+^. Intracellularly, we detected major differences in the abundance and subcellular distribution of granzymes, perforin, and granulysin. Similar differences were seen in enriched lysosome-related effector vesicles (LREVs). The proteomics profiling of enriched EVs from an NK-LGLL line (NKL) and a T-LGLL line (MOTN-1), confirmed individual profiles of effector molecules. Conclusion: Our analyses underscore the individual distribution of effector proteins but also open new routes to define the role of intra- and extracellular granules in the disease manifestation or pathology of LGLLs.

## 1. Introduction

Large granular lymphocyte leukemia (LGLL) is a rare chronic lymphoproliferative disorder with heterogeneous clinical presentation. It is characterized by clonal expansion of lymphocytes with granulated cytoplasm [1]. The fifth edition of the WHO classification of hematolymphoid tumors places LGLL in the category of mature T-cell and NK cell leukemias with three major subtypes: T-large granular lymphocytic leukemia (T-LGLL), NK-large granular lymphocytic leukemia (NK-LGLL), and aggressive NK-cell leukemia (ANKL). A further distinction can be made between CD4^+^ or CD8^+^ T-LGLL and αβ- or γδ-T-LGLL [2]. LGL leukemia accounts for approximately 2–5% of chronic lymphoproliferative diseases in the US and Europe [1]. The incidence is reported to be 0.2–0.72 per one million per year [3,4]. The median age of onset is 60 years and both sexes are similarly affected [5]. Chronic T-LGLL accounts for 85% of cases, while NK-LGLL accounts for less than 10%. Aggressive NK-LGL leukemia (ANKL) occurs more frequently in Asia and affects slightly younger patients, particularly those with a history of EBV infection [6].

Diagnosis and characterization of LGLL-subtypes are based on cytology, immunophenotype, and evidence of monoclonality [7]. The phenotype of T-LGLL is heterogeneous, being CD2^+^, CD3^+^, CD8^+^, CD57^+^, CD45RA^+^, CD16^+^, and CD4^−^, with or without expression of CD56 [7,8]. As mentioned, αβ-TCRs are often expressed, but γδ-TCR^+^ cases have also been described [6,9,10]. Overall, this T-cell phenotype corresponds to a constitutively active mature memory T effector cell [8]. In T-LGLL clones, cytotoxic effector proteins including granzymes and perforin are frequently expressed, although in different combinations. A less frequent subtype occurs in 10–15% of cases and is CD4^+^, CD57^+^, CD8^−^ or CD8^+dim^ [9]. NK-LGLL are mostly CD2^+^, CD3^−^, CD8^+^, CD16^+^, CD56^+^ and also express cytotoxic effector molecules [8].

Although significant progress has been made in our understanding of genetic and molecular alterations associated with LGLL (see recent overviews [1,9,11,12,13,14] for details), the contribution of the LGLL-associated cytotoxicity and respective effector molecules has only been poorly addressed. Initially, it became evident that dysregulated apoptosis may represent a key event in the development of LGL malignancy and autoimmunity. Perzova and Loughran reported that seven out of the seven LGLL patients tested displayed constitutive expression of FasL gene transcripts [15]. It was then observed that leukemic cells from 9 out of 11 T-LGLL patients constitutively expressing both Fas and FasL at high levels were completely resistant to anti-Fas- and anti-CD3-mediated apoptosis [16]. Interestingly, the LGLL T cells did not show apparent mutations in Fas and in most cases underwent apoptosis after PHA and IL-2 stimulation [16]. Over the years, FasL was also associated with other LGLL symptoms, including neutropenia. The serum level of (soluble) sFasL, which is involved in preventing Fas-mediated apoptosis, was proposed as a marker for LGLL activity [17,18].

For other cytotoxic effectors, data pointing to dysregulation are hardly available. In fact, granzyme (Grz) B and perforin (Prf) are often mentioned as additional markers to ascertain a diagnosis of LGLL, especially for histological staining of bone marrow biopsies [5]. Surprisingly for granulysin (Gnly), another key effector of T and NK cells, the distribution, release, or impact in LGLL has apparently never been addressed. The presence of elevated levels of sFasL in LGLL patients’ sera might, however, indicate a substantial production and release of cytotoxic granules from leukemic cells. Although sFasL has been correlated with the prevention of apoptosis in leukemic cells, the exposure or release of vesicles carrying the death ligand might have broader implications. In particular, the unsafe exposure of molecules transported in secretory vesicles or a release of vesicles carrying effectors as extracellular vesicles might contribute to immune dysfunction and the development of autoimmune diseases.

As a first attempt to tackle these issues, we addressed the distribution and release of cytotoxic effector molecules in established LGLL cell lines. Previously, we compared intracellular effector vesicles from the patient-derived NK LGLL line NKL [19] with those from the NK cell lymphoblastic leukemia/lymphoma line YTS [20,21] and from untransformed cells. We reported heterogeneous profiles of functionally relevant proteins including cytotoxic effector proteins in leukemic and activated human NK cells [22].

We now followed this route and analyzed patient-derived LGLL cells, in particular MOTN-1 [23], representing a T-cell LGLL; NKL [19] and NK-92 [24], representing two NK-LGLLs; and KHYG-1 [25], representing an ANKL. For those cell lines, all individual cellular characteristics have been described in detail before [19,23,24,25]. As stated above, individual LGLL subtypes differ in the expression of lineage-specific surface markers, display different mutations in genes of either the STAT3/5B or the NOTCH1 pathway, and might produce different immunomodulatory cytokines.

Interestingly, in our hands, all cell lines did express comparable levels of CD26, but did not express (membrane) mFasL. When we analyzed the distribution of effector proteins by conventional and imaging flow cytometry or Western blotting, we detected major differences in the abundance of granzyme A, granzyme B, perforin, and granulysin. Similar differences were also seen in cytotoxic granules enriched as lysosome-related effector vesicles (LREVs). In untransformed T and NK cells, such LREVs, previously also described as secretory lysosomes [26], form the basis for a differential release of individual cytotoxic effector proteins depending on the strength of stimulation [27]. As an example, we demonstrated that granulysin (Gnly) species might be used as markers for distinct entities of lysosome-related effector vesicles (LREVs) since in untransformed T-cell blasts, they segregate to different secretory compartments with granzyme B or FasL and become mobilized by either classical or non-classical degranulation [28].

Due to a potential relation of LREVs or secretory lysosomes to extracellular vesicles released as exosomes or microparticles [27], we performed a comparative proteome profiling of isolated EV from MOTN-1 and NKL to obtain a deeper insight into individual proteins’ composition and to address the potential role of LREVs as intra- and extracellular components for disease manifestation or pathology.

## 2. Materials and Methods

### 2.1. Cell Lines

Notably, only a few established cell lines are available for the study of NK-LGLL biology [29,30]. For our studies, we used NK-92 (ACC 488), which was established in 1992 from the peripheral blood of a 50-year-old male with LGL-non-Hodgkin lymphoma [24] and NKL (CVCL_0466), which was established in 1996 from the peripheral blood of a 63-year-old patient with a more aggressive CD3^−^, CD16^+^, and CD56^+^ LGL leukemia [19]. Both cell lines are now categorized as NK-LGLL and are supposed to display strong cytotoxicity. This also holds true for KHYG-1 (ACC 725), an ANKL cell line established in 1997 from the peripheral blood of a 45-year-old woman with aggressive NK leukemia [25]. The T-LGLL line MOTN-1 (ACC 559) was established in 2001 from the peripheral blood of a 65-year-old woman with chronic T-LGLL; in this case, no cytotoxic activity for expanded cells was described [23]. All cell lines were kept at 37 °C in a humidified atmosphere with 5% CO_2_. The culture medium was RPMI 1640 with 2 mM glutamine and 25 mM HEPES (Thermo Fisher Scientific, Waltham, MA, USA), supplemented with 100 U/mL penicillin/100 μg/mL streptomycin (Thermo Fisher Scientific) and 10% (*v*/*v)* fetal calf serum (FCS, Thermo Fisher Scientific) for NKL and KHYG-1 or 20% (*v*/*v*) FCS for MOTN-1. NK-92 cells were cultured in Minimal Essential Medium α (MEM α, #32561029, Thermo Fisher Scientific) with 12.5% (*v*/*v*) horse serum (Thermo Fisher Scientific) and 12.5% (*v*/*v*) FCS. Since growth of LGLL cell lines is strictly IL-2-dependent, recombinant IL-2 (rIL-2, Novartis, Basel, Switzerland) was added at 50 U/mL for NKL, 100 U/mL for NK-92 and MOTN-1, and 200 U/mL for KHYG-1 expansion.

### 2.2. Immunofluorescent Staining

FasL and CD26/DPP4 surface expression was checked by conventional flow cytometry [31]. A total of 10^5^ cells were centrifuged in 96-well V-bottom plates, washed, and stained with PE-conjugated anti-FasL monoclonal antibody (mab) (clone NOK-1, Fisher Scientific) or anti-CD26/DPP4 mab (clone BA5b, BioLegend, San Diego, CA, USA). After incubation for 30 min on ice, the cells were washed twice, fixed in 1% paraformaldehyde, and analyzed on a FACS Canto flow cytometer (BD Biosciences, Franklin Lakes, NJ, USA). For staining of intracellular antigens by flow cytometry, cells were washed, fixed, and permeabilized using the Cytofix/Cytoperm kit (BD-Biosciences) according to the manufacturer’s instructions. After fixation, the cells were analyzed on an FACS Canto flow cytometer. The following antibodies were used: PE-conjugated anti-granzyme A mab (clone CB9, BioLegend), PE-conjugated anti-granzyme B recombinant ab (clone QA16A02, BioLegend), PE-conjugated anti-perforin mab (clone dG9, BioLegend). For granulysin staining, we also used an unconjugated anti-Gnly mab (clone RF10, MBL International, Woburn, MA, USA) with Alexa-Fluor 555-conjugated goat anti-mouse polyclonal antibody (pab) (Thermo Fisher Scientific) and unconjugated anti-Gnly pab from goat (RD Systems, Minneapolis, MN, USA) with Alexa-Fluor 555-conjugated donkey anti-goat pab, respectively. PE-conjugated isotype-matched antibodies from BioLegend and unconjugated antibodies from Abcam (Cambridge, UK) served as controls [28].

For imaging flow cytometry, 0.5 × 10^6^ cells were washed and suspended in 100 µL LIVE/DEAD Fixable Far-Red stain (Thermo Fisher Scientific) for 15 min, washed again, and permeabilized in 50 µL of Cytofix/Cytoperm. After 30 min, the cells were washed and incubated with a FITC-conjugated anti-CD107a mab (clone H4A3, BioLegend) for another 30 min. Two additional washes were followed by addition of PE-conjugated anti-granzyme A, anti-granzyme B, anti-perforin, or anti-CD107a as a control. For granulysin staining, we also used unconjugated anti-Gnly mab RF10 or unconjugated anti-Gnly pab with Alexa-Fluor 555-conjugated secondary reagents and modified the staining procedure accordingly. Finally, cells were fixed in 1% paraformaldehyde.

### 2.3. Imaging Flow Cytometry

Imaging flow cytometry was performed exactly as described before with an ImageStream X Mark II (Merck Millipore, Burlington, MA, USA) one-camera system with 351, 488, 562, 658, and 732 nm lasers [31]. Analyte-positive cells were discriminated based on controls. The Bright Detail Similarity R3 feature was used to quantify the degree of colocalization in double-positive cells only [31].

### 2.4. Subcellular Fractionation

Lysosome-related effector vesicles (LREVs) were enriched from expanded cell lines as described before [28,32], employing a commercial lysosome isolation kit (Merck/Sigma-Aldrich) and differential centrifugation and ultracentrifugation.

### 2.5. Immunoprecipitation and Western Blot

To obtain cell lysates for Western blotting, 10 × 10^6^ cells were washed in PBS and lysed in NP40 lysis buffer (1% (*v*/*v*) Nonidet^®^P40 (Sigma-Aldrich), 20 mM Tris-buffer, pH 7.4, 150 mM NaCl, 5 mM EDTA) supplemented with protease and phosphatase inhibitors for 30 min [28]. Cell debris was removed by centrifugation at 14,000× *g* rpm and 4 °C for 10 min and supernatants were boiled in reducing sample buffer. To precipitate granulysin from supernatants of unstimulated or stimulated LGLL cells, 10 × 10^6^ cells were washed in PBS and suspended in X-vivo medium (Thermo Fisher Scientific). TPA and/or ionomycin (both from Merck) were added at 20 or 500 ng/mL, respectively, to stimulate the cells for up to 2 h in the presence or absence of 4 mM EGTA and 4 mM MgCl_2_. Following stimulation, the cells were pelleted, washed with PBS, and lysed in NP40 lysis buffer. Supernatants were centrifuged at 3400× *g* to remove residual cells and subjected to immunoprecipitation. To this end, NP-40 lysates or supernatants were precleared with protein G-sepharose beads (Sigma-Aldrich) for 2 h and incubated overnight with 0.5 μg of the polyclonal anti-Gnly antibody (R&D Systems Inc., Minneapolis, MN, USA) and protein G-sepharose beads at 4 °C. After three washes, samples were boiled in reducing sample buffer and subjected to gel electrophoresis on Bis-Tris NuPAGE gels (Thermo Fisher Scientific). Proteins were transferred to nitrocellulose membranes (GE Healthcare, Munich, Germany) and membranes were blocked with bovine serum albumin (Sigma-Aldrich) in TBST (5%, *w*/*v*). The polyclonal anti-Gnly antibody was then used for detection with HRP-conjugated donkey anti-goat IgG antibodies (Abcam) and ECL chemiluminescence reagents and Hyper Film (GE Healthcare).

### 2.6. Generation of Extracellular Vesicles for Proteome Analyses

To compare extracellular vesicles from T and NK-LGLL cells, we focused on NKL and MOTN-1. First, 4 × 10^8^ cells were washed twice with PBS before expansion and cultivation for 72 h in 100 mL exosome-reduced culture medium in the presence of rIL-2. To deplete exosomes, RPMI 1640 medium supplemented with 20% FCS and penicillin/streptomycin was centrifuged at 4 °C and 100,000× *g* (Beckman XL-80 centrifuge, SW 32.1 Ti rotor, Krefeld, Germany) for 18 h. For NKL cell cultures, the EV-depleted supernatant was filtered and further diluted with serum-free RPMI 1640 medium containing penicillin/streptomycin to a final concentration of 10% FCS. Particle concentrations in exosome-depleted culture media were occasionally tested via nanoparticle tracking analysis, as detailed before [33].

Extracellular vesicles (EVs) were isolated by differential centrifugation and ultracentrifugation as described in [33]. Briefly, culture supernatants of NKL or MOTN-1 cells kept in EV-reduced medium were centrifuged for 10 min at 300× *g*, 30 min at 2000× *g* and for 45 min at 10,000× *g* to remove intact cells and cell debris. EVs were pelleted by ultracentrifugation at 100,000× *g* for 1.5 h, washed once with filtered (0.1 nm) PBS at 100,000× *g* for 1.5 h, and resuspended in 0.3 mL filtered PBS. EVs’ size and concentration were determined via nanoparticle tracking analysis (NTA) with a NanoSight 300 (NS300) using NanoSight software version 3.40 (Malvern Panalytical, Malvern, UK).

### 2.7. LC-MS-Based Proteome Analysis

EV samples were provided in 100 µL PBS buffer, and SDS was added to a final concentration of 1% *w*/*v*. Samples were lysed in a Bioruptor Pico (Diagenode, San Diego, CA, USA) at 4 °C (10 × 30 s on, 30 s off) and 20 µL was used for BCA analysis (Pierce BCA Protein Assay Kit, Thermo Fisher Scientific) according to the manufacturer’s protocol (MOTN-1: 0.255 µg/µL, NKL: 0.224 µg/µL). Per sample, 10 µg protein was subjected to reduction and alkylation (12 mM Tris(2-carboxyethyl)phosphine, 40 mM 2-chloroacetamide for 1 h at 25 °C) and desalting performed according to the SP3 protocol [34]. SP3 beads were added to each sample (1:20 *w*/*w* protein to beads ratio) and ethanol was added to 50% *v*/*v* to induce protein binding (15 min at 25 °C). Beads were immobilized using a magnetic rack, the supernatants were discarded, and the beads washed three times with 80% *v*/*v* ethanol. The pellets were resuspended within 50 mM TEAB buffer (pH 8.5) with 0.2 µg trypsin (1:50 enzyme to protein ratio, Promega, Madison, WI, USA), incubated for 16 h at 37 °C, and the supernatants were acidified using trifluoroacetic acid (TFA) for LC-MS/MS measurement.

Samples were injected in triplicate on a Dionex Ultimate 3000 nano-UHPLC coupled to a Q Exactive mass spectrometer (Thermo Fisher Scientific). Per injection, 1 µg protein was loaded onto a trap column (Acclaim Pepmap 100 C-18, 5 mm × 300 μm, 5 μm, 100 Å, Dionex, Sunnyvale, CA, USA) and washed for 5 min with 3% ACN/0.1% TFA at a flow rate of 30 μL/min prior to peptide separation using an Acclaim PepMap 100 C-18 analytical column (50 cm × 75 μm, 2 μm, 100 Å, Dionex). A flow rate of 300 nL/min using eluent A (0.05% formic acid (FA)) and eluent B (80% ACN/0.04% FA) was used for gradient separation (5–50% eluent B). A spray voltage of 1.5 kV was applied via a liquid junction emitter (MS Wil Fused silica emitter, CoAnn Technologies LLC, Richland, WA, USA), with a source temperature of 250 °C. Full scan MS spectra were acquired between 300 and 1800 *m*/*z* at a resolution of 70,000 at *m*/*z* 200, and top ten most intense precursor ions were selected for MS/MS analysis (charge state: ≥+2, isolation window: ±1.5 *m*/*z*, HCD fragmentation: 27 NCE). MS/MS spectra were acquired at a resolution of 17,500 with fixed first mass at *m*/*z* 100, lock mass enabled (*m*/*z* 445.120025), and a dynamic exclusion list of 40 s.

MS raw files were processed by Proteome Discoverer (version 2.2.0.388, Thermo Fisher Scientific) using the Sequest HT algorithm against a human protein database with additional common contaminating proteins (only reviewed sequences) from the UniProt database [35]. The enzymatic processing was set to trypsin (full-specific); maximum missed cleavage events: 2; minimal peptide length: 6 AA; precursor mass tolerance: 10 ppm; fragment mass tolerance: 0.02 Da; and false-discovery rate set to q = 0.01 by a Percolator node [36]. Label-free quantification was performed by a Minora feature detector with a subsequent feature mapper (minimum trace length: 5; maximum RT shift: 4 min; mass tolerance: 10 ppm; parameter tuning: fine). Three technical injections were used as replicates for the quantification strategy and data filtered for high-confidence proteins with a minimum of two peptides/one unique peptide identification. Abundance values were median normalized and statistical testing was performed by a two-sided Welch t-test with Benjamini-Hochberg FDR calculation (4e = 0.01) in Perseus [37]. GO annotations were added and 1D enrichment analysis and Fisher’s exact test were performed with a 5% FDR level. All proteomics raw data have been uploaded to the ProteomeXchange Consortium [38] via the PRIDE partner repository with the dataset identifier PXD053228.

## 3. Results

### 3.1. Phenotypic Peculiarities of LGLL Cell Lines Analyzed by Flow Cytometry

**Figure 1:**
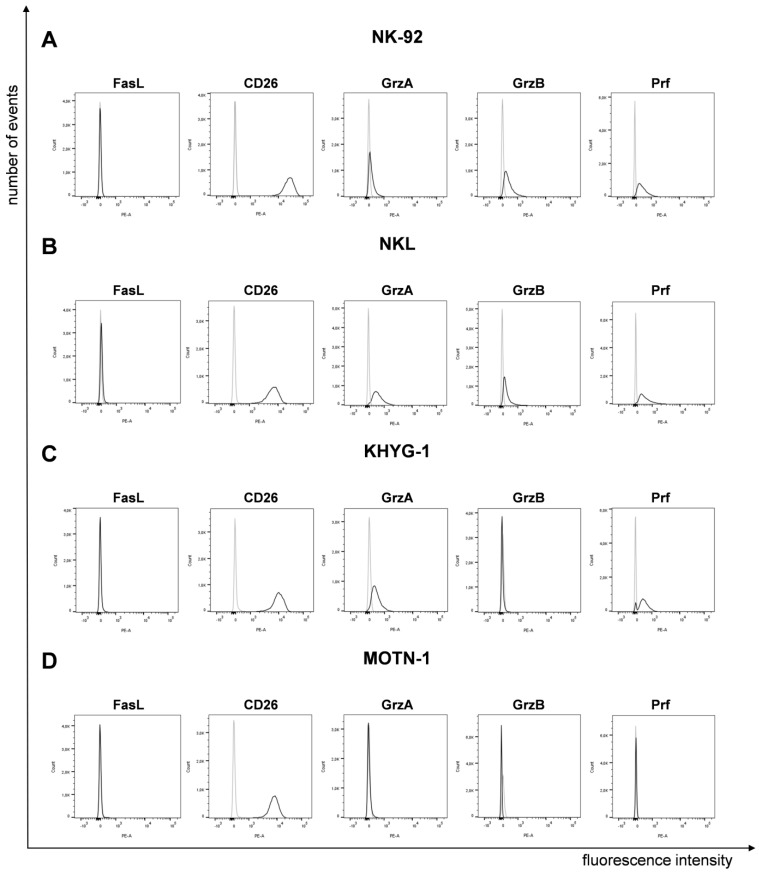
Expression of FasL, CD26 and cytotoxic effector proteins on/in LGLL cells. Analysis of surface FasL and CD26 and intracellular GrzA, GrzB and Prf on/in NK-92 (**A**), NKL (**B**), KHYG-1 (**C**) and MOTN-1 (**D**). First, 10^5^ cells were stained directly with PE-conjugated mAb against FasL (NOK1, IgG1) and CD26 (BA5b, IgG2a) or after permeabilization with Cytofix/Cytoperm, with PE-conjugated mAb against GrzA (CB9, IgG2b), GrzB (QA16A02, IgG1), and Prf (dG9, IgG2b) or with respective isotype control antibodies. Stained cells were analyzed after fixation on a BD FACS Canto flow cytometer. One representative experiment out of three is shown.

It was reported that LGLL cells display a characteristic surface decoration of lineage-specific markers [7] and a constitutive expression of functionally dysregulated FasLigand (FasL, CD95L, CD178) and Fas (CD95) [15,16]. However, according to our analyses, the four well-characterized cell lines (NK-92, NKL as NK-LGLL, KHYG-1 as ANKL and MOTN-1 as T-LGLL) used in the present study did not display any (membrane) mFasL, but high amounts of CD26 (DPP4), which we had previously associated with the degranulation of lysosome-related effector vesicles (LREVs) [31] (Figure 1).

To analyze the presence of cytotoxic effector molecules, we permeabilized the cells for intracellular staining of granzymes (Grz) A and B and perforin (Prf). We used PE-conjugated monoclonal antibodies as specified before or respective isotype control antibodies (Figure 1). The two NK-LGLLs, NK-92 and NKL, were positive for all effectors, with a slightly higher fluorescence intensity for GrzA in NKL. In KHYG-1, we detected GrzA and Prf, but no GrzB. As expected from the characterization of MOTN-1, not showing any cytotoxic activity [23], we did not detect any of the tested effector proteins by intracellular staining (Figure 1).

### 3.2. Analysis of Granulysin and Granzyme B in Enriched LREVs

**Figure 2:**
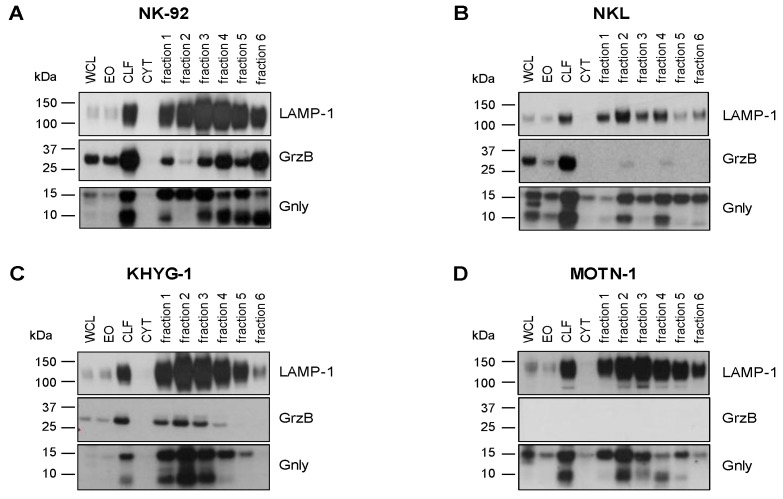
Cytotoxic effector proteins in LGLL cells. Analysis of internal GrzB and Gnly in NK-92 (**A**), NKL (**B**), KHYG-1 (**C**) and MOTN-1 (**D**). Cells were mildly homogenized with a balch homogenizer and subjected to differential and density centrifugation as described. Whole cell lysate (WCL), enriched organelles (EO), crude lysosomal fraction (CLF), and the cytosol (CYT) were separated together with fractions 1–6 of the density gradient on Bis-Tris NuPAGE gels, transferred to nitrocellulose, and tested for the presence of LAMP-1 and GrzB with respective mab from BD Bioscience and BioLegend and HRP-conjugated anti-mouse IgG antibodies from Cytiva. Gnly was stained with a polyclonal goat anti-Gnly antibody from R&D followed by HRP-conjugated anti-goat IgG antibodies from Abcam.

We have shown before that granulysin (Gnly) species might be used as markers for distinct entities of lysosome-related effector vesicles (LREVs) since, in untransformed T-cell blasts, they segregate to different secretory compartments and become mobilized by either classical or non-classical degranulation [28]. In order to characterize the distribution of LREVs in LGLL cells, we thus analyzed enriched LREVs [32] for the presence of Gnly, GrzB, and LAMP-1 (Figure 2). To our surprise, the Western blot results were quite heterogeneous and indicated a differential distribution of LREVs carrying individual effector molecules. In all cells, we detected LAMP-1 as a marker for lysosomes in the complete lysosomal fraction and in all individual fractions after density gradient and ultracentrifugation. Notably, in NK-92 cells, most LAMP-1 was detected in fractions 3 and 4, whereas in the other preparations, more LAMP-1 was detected in fractions 2 and 3. The distribution of GrzB and Gnly was quite different in the individual cell lines. GrzB was not detected in MOTN-1 but was present in the crude lysosomal fractions of the two NK-LGLLs and the ANKL cell lines. However, the subcellular distribution of GrzB differed in NK-92 and NKL or KHYG-1. Whereas in NK-92, GrzB was enriched in heavy fractions and especially in fraction 6 vesicles, it was much less intense in KHYG-1 and hardly detectable in NKL LREVs. For Gnly, the patterns seen in NK-92 corresponded to what has been described for untransformed T cells [28]. The 9 kDa Gnly, representing the mature form, was detected primarily in heavier fractions associated with GrzB, whereas the 15 kDa form was more abundant in the lighter fractions 1 to 3. Notably, both Gnly species were stained in the crude lysosomal fraction of all four cell lines. Interestingly, in KHYG-1 and in NKL, although in distinct fractions, both 9 and 15 kDa Gnly were associated with GrzB. In NKL, KHYG-1 and MOTN-1, 9 kDa Gnly, however, was primarily associated with the lighter fractions 1 to 4. Given the high similarity in protein distribution in LREVs from untransformed cells, these results might indicate that subcellular sorting and distribution of effector proteins are altered in LGLL cells.

### 3.3. Imaging of the Intracellular Distribution of Cytotoxic Effector Proteins

**Figure 3:**
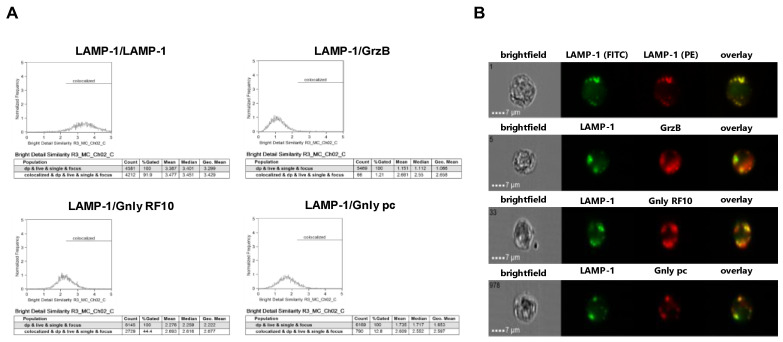
Colocalization of intracellular LAMP-1 with GrzB and the 15 kDa (RF10) or 9 kDa (pc) form of Gnly in NK-92 cells. Following fixation and permeabilization, cells were stained with FITC-conjugated anti-LAMP-1 mab. After washing, cells were additionally stained with PE-conjugated anti-LAMP-1 mab (as a positive control for colocalization), PE-conjugated anti-GrzB mab, or with anti-Gnly mab RF10 or a polyclonal anti-Gnly pab (pc) and appropriate Alexa Fluor 555-conjugated secondary antibodies. A total of 10,000 cells were acquired with an ImageStream Mark II imaging flow cytometer. Only focused, single cells were considered for further analyses. (**A**) Histograms display the geometric mean value of the BDS score of respective stainings and the percentage of cells displaying a BDS score >2. (**B**) Representative images of stained cells. Scale bars represent 7 µm.

**Figure 4:**
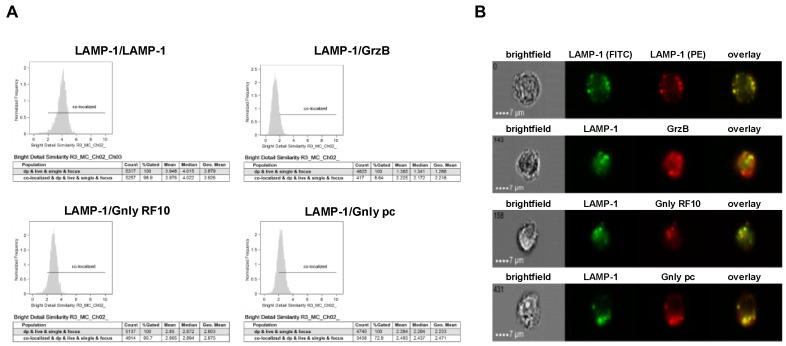
Colocalization of intracellular LAMP-1 with GrzB and the 15 kDa (RF10) or 9 kDa (pc) form of Gnly in NKL cells. Samples were processed as detailed in the legend of Figure 3. (**A**) Histograms display the geometric mean value of the BDS score of respective stainings and the percentage of cells displaying a BDS score >2. (**B**) Representative images of stained cells. Scale bars represent 7 µm.

We next employed imaging flow cytometry to analyze the subcellular localization of LAMP-1, GrzB, and Gnly in NK-92 and NKL cells (Figure 3 and Figure 4). Notably, almost all cells stain positive for LAMP-1 (NK-92 97.7%, NKL 99.4%), and 97.1% of NK-92 cells and 97.3% of NKL cells stain positive for GrzB. To differentiate between the 15 and 9 kDa forms of Gnly, we employed established Gnly detection reagents with differential binding properties. The monoclonal antibody (mab) RF10 exclusively binds to the full-length 15 kDa variant, whereas the polyclonal antibody (pab) termed pc almost exclusively recognizes the 9 kDa form in PFA-fixed samples [28,39]. The 15 kDa variant of Gnly is expressed by only 3.2% of NK-92 cells and 83.1% of NKL cells, whereas the 9 kDa form is found in 28.7% of NK-92 cells and 54.3% of NKL cells. We also quantified the degree of colocalization of cytotoxic effector proteins with LAMP-1 in double-positive cells in NK-92 (Figure 3) and NKL (Figure 4) cells.

As expected, LAMP-1 is located in granular structures within both NK-92 and NKL cells and, as a positive control, nearly all cells display a bright detail similarity score >2 for the colocalization of FITC- and PE-labeled anti-LAMP-1 ab. Surprisingly, the cytotoxic effector protein GrzB hardly colocalizes with LAMP-1 since less than 10% of double-positive NKL and hardly any NK-92 cells show a BDS score >2 for the GrzB/LAMP-1 costaining. In NK-92 cells that express both the 15 and the 9 kDa form of Gnly, only about 10% of double-positive cells store 9 kDa Gnly in LAMP-1-positive intracellular granula, whereas 44% of the cells display a BDS >2 for RF10/LAMP-1-costainings, indicating a storage of 15 kDa Gnly in LAMP-1^+^ LREVs (Figure 3). Notably, in Gnly^+^ NKL cells, both the 15 and 9 kDa form colocalize with LAMP-1, with the 15 kDa Gnly/LAMP-1 colocalization being more pronounced (Figure 4). Thus, compared to untransformed cytotoxic CD8^+^ αβ-TCR^+^ and to γδ-TCR^+^ T cells where granzymes and the 9 kDa Gnly but usually not the 15 kDa Gnly associate with intracellular LAMP-1^+^ granular structures, the LGLL cells analyzed here apparently have a more heterogeneous and altered effector protein storage machinery.

### 3.4. Differential Activation-Induced Release of 9 or 15 kDa Granulysin

**Figure 5:**
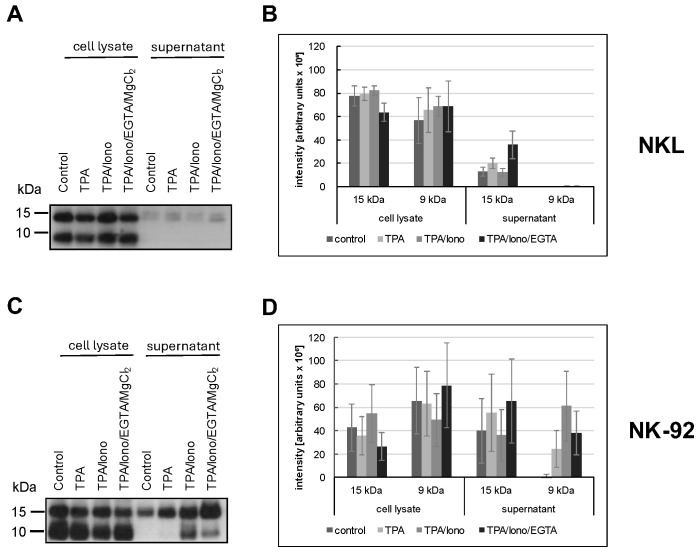
NKL (**A**,**B**) or NK-92 (**C**,**D**) cells were stimulated or not for two hours with TPA and/or ionomycin with or without EGTA. The presence of 15 kDa and 9 kDa Gnly was assessed in cellular lysates and in precipitations with anti-Gnly (pc) from supernatants of unstimulated or stimulated cells. The blot was stained with the anti-Gnly (pc) to detect both forms of Gnly, as detailed in the Results section. (**A**,**C**) Representative Western blots. (**B**,**D**) Densitometric analysis of three independent experiments as depicted in (**A**,**C**) for 9 kDa Gnly and 15 kDa Gnly. Data are displayed as mean values ± standard deviation.

We previously reported that in untransformed T cells, individual LREVs containing either 15 kDa or 9 kDa Gnly utilize different routes for mobilization and release referred to as Ca^2+^-independent non-classical and Ca^2+^-dependent classical degranulation, respectively [40]. Given the heterogenous and altered effector protein storage patterns in LGLL cells, we next analyzed the signal requirements for the release of the two forms of Gnly. The release of Gnly into cell culture supernatants was addressed by immunoprecipitation and Western blotting after stimulation with phorbolester (TPA, non-classical degranulation) or phorbolester and calcium ionophore (TPA/ionomycin, classical degranulation) in the absence or presence of the Ca^2+^ chelator EGTA (Figure 5).

We used the polyclonal anti-Gnly antibody (pc) as a primary antibody for Gnly detection in Western blots after immunoprecipitation of Gnly from supernatants to determine which form of Gnly is secreted into the culture supernatant upon stimulation with either phorbol ester and/or calcium ionophore. NKL cells hardly released any Gnly, although both forms were apparently expressed, as evidenced by the Western blot of whole cell lysates in Figure 5A. In contrast, we readily precipitated Gnly with the polyclonal anti-Gnly (pc) antibody from culture supernatants of unstimulated or TPA- and TPA/ionomycin-stimulated NK-92 cells. TPA activation resulted in an increased and selective release of the 15 kDa variant into the culture supernatant, whereas TPA in combination with ionomycin also induced the release of the 9 kDa form. Importantly, this Ca^2+^-dependent release triggered by TPA and ionomycin could be partially abrogated by Ca^2+^-chelation with EGTA. These results again highlight the more heterogenous and altered storage and release patterns of cytotoxic effector proteins in LGLL cells.

### 3.5. Proteomics Analysis of NKL- and MOTN-1-Derived Extracellular Vesicles

**Figure 6:**
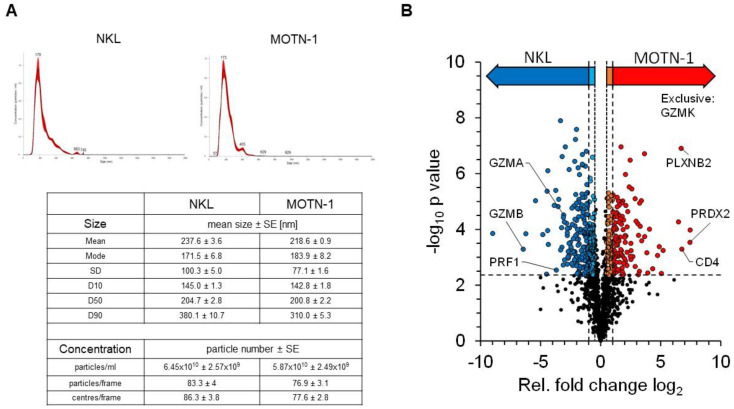
(**A**) Characteristics of enriched extracellular vesicles from NKL and MOTN-1. Extracellular vesicles (EVs) were enriched by ultracentrifugation as detailed in the Methods section and subjected to nanoparticle-tracking analyses using a NS300 device and NanoSight software version 3.40 from Malvern Panalytical. Graphs for particle distribution are shown and key results comprising particle size and concentration are listed. (**B**) Quantitative proteomics analysis of EV samples derived from NKL and MOTN-1 cells. The volcano plot highlights the relative fold changes of all quantified proteins with the corresponding significance values. The horizontal line represents the statistical cutoff of q = 0.01 and the vertical lines represent the biologically relevant fold changes (outer lines: strict filter, inner lines: moderate filter). Two-sided Welch *t*-test with Benjamini-Hochberg FDR calculation; *n* = 1174 proteins.

We previously reviewed the close relationship between intra- and extracellular effector vesicles in T and NK cells [27]. To characterize and compare the protein load of extracellular vesicles (EV) from T- and NK-LGLL cell lines, a bottom–up proteomics analysis was performed with constitutively produced EV from MOTN-1 and NKL. To this end, 4 × 10^8^ cells were washed with PBS before expansion and cultivation for 72 h in 100 mL exosome-reduced culture medium in the presence of rIL-2. EVs were collected and enriched as described and characterized by nanoparticle-tracking analyses (NTAs). The final concentration of particles was comparable in both preparations (6.45 × 10^10^ ± 2.59 × 10^9^ particles/mL for NKL and 5.87 × 10^10^ ± 2.49 × 10^9^ particles/mL for MOTN-1), as was the size of the enriched EVs (mean: 237.6 ± 3.6 nm for NKL and 218.6 ± 0.9 nm for MOTN-1; mode: 171.5 ± 6.8 nm for NKL; and 183.9 ± 8.2 nm for MOTN-1). As depicted in Figure 6A, the EV/exosome preparations were quite homogeneous, containing mainly small extracellular vesicles (sEVs) of around 200 nm in diameter and only minor fractions of larger vesicles.

The proteomics analysis of the samples in three technical injections revealed 1742 high-confidence protein identifications, of which 1174 proteins were quantifiable (see Supplementary Table S1 for comprehensive information). Exosome proteins were markedly enriched within the dataset, with 766 proteins being associated with the GO term “extracellular exosomes” (GO:0070062). These included common marker proteins such as the tetraspanins CD63 and CD81, as well as other EV-related proteins such as syntenin-1, TSG101 or the programmed cell death 6-interacting protein/Alix. Notably, the tetraspanin CD9 was missing in the dataset and was not identified in any sample (Table 1).

Other proteins which are typically present in EVs of different cell types include flotillins, annexins, and heat shock proteins. Several members of these protein families were also detected in EVs from NKL and MOTN-1. Moreover, cell adhesion molecules such as ICAM-1 (CD54), VCAM-1 (CD106) and DNAM-1 (CD226) as well as various integrins (integrin α-4, integrin β-1, -2, -7) were detected. The list of identified proteins also includes several Rab GTPases associated with vesicle transport (such as RAB5B, RAB7A, RAB11B, and RAB27A), ADAM proteases that are known to be associated with intra- and extracellular vesicles (such as ADAM8 and ADAM10), and lysosome-associated membrane proteins LAMP-1 (CD107a) and LAMP-2 (CD107b) (Table 1). In addition, numerous transmembrane or cytosolic receptors and signaling proteins and various nuclear or ribosomal proteins were identified in the enriched EVs (Supplementary Table S1).

Quantifiable proteins were subjected to statistical testing by a two-sided Welch t-test to identify proteins with altered abundance levels between both samples (Figure 6B). Relative differences were considered biologically relevant at a 2-fold difference (log_2_(FC) ≥ 1 or ≤−1, strict filter) or at a 1.4-fold difference (log_2_(FC) ≥ 0.5 or ≤−0.5, moderate filter), both including statistical significance at q = 0.01. This analysis provided 186 proteins with significantly higher abundance levels in MOTN-1 samples (strict: 119, moderate: 67), as well as 263 proteins with higher abundance levels in NKL samples (strict: 173, moderate: 90). The MS analysis also identified 34 proteins, which were exclusively identified within MOTN-1 samples and 21 exclusively in NKL samples (Supplementary Table S2).

Proteins with higher abundance levels in NKL-derived EVs included, for example, granzyme B (*GZMB*), Zyxin (*ZYX*), and the ubiquitin-like protein ISG15 (*ISG15*), but also other cytotoxic effector proteins including perforin (*PRF1*) and granzyme A (*GZMA*). MOTN-1-derived EVs presented more peroxiredoxin-2 (*PRDX2*), the T-cell surface glycoprotein CD4 (*CD4*), plexin-B2 (*PLXNB2*), and TNF-receptor 2 (*TNFRSF1B*) (Table 2). Interestingly, granzyme K (*GZMK*) was instead exclusively identified in EVs of the T-LGLL MOTN-1. The proteins with the largest fold changes (FC log_2_) within both samples were the beta chains of the major histocompatibility complex HLA-DR: MOTN-1 contained DRB1–8 with a log_2_(FC) of 7.45, while NKL contained DRB1-1 with log_2_(FC) of −8.99. Both proteins provide a high sequence homology, with the MS-based quantification being restricted to a few sequence-unique peptides.

Notably, LAMP-1 showed increased abundance levels in MOTN-1 samples, whereas no significant difference in protein abundance was seen for LAMP-2. Further, the EV-associated dipeptidyl peptidase 4 (DPP4, CD26) was also detected without significant difference in protein abundance in both types of EVs. The activating NK cell receptors “Natural killer cell receptor 2B4” and “Killer cell immunoglobulin-like receptor 2DL4 (*KIR2DL4*)” were identified in EVs of both NKL and MOTN-1 without significant fold changes, while the inhibitory receptor “immunomodulatory receptor leukocyte immunoglobulin-like receptor B1” was detected with a higher abundance level in NKL. The death receptor Fas was detected in EVs of both cell lines; however, its abundance was significantly increased in the MOTN-1-derived vesicles (Table 1).

For further characterization of the proteins, GO terms were linked to each individual protein and those were analyzed by Fisher’s exact test and 1D enrichment analysis (Supplementary Tables S3 and S4). The Fisher’s exact test uses the enrichment factor (EF) to test the enrichment of protein groups among those with significant changes between MOTN-1 and NKL. In EV of MOTN-1, significantly enriched GO terms were detected as chaperonin-containing T-complex and protein folding, both terms subsuming proteins associated with protein folding. In addition, the terms sperm–egg recognition and binding of sperm to zona pellucida were enriched, as well as the term antigen presentation. Proteins associated with membranes (external side of plasma membrane, intrinsic to endoplasmic reticulum membrane, and integral to the organelle membrane) were also increased in MOTN-1. In EVs of NKL, the following terms were detected as significantly enriched: protein–DNA complex and nucleosome, which include proteins that interact with DNA and are involved in its packaging, e.g., histones. In addition, terms that can be attributed to the cytoskeleton and cell movement were enriched in the EVs of NKL cells. For the proteins mentioned in Table 1 and Table 2, GO-terms are summarized in Supplementary Table S5.

## 4. Discussion

Large granular lymphocyte leukemia (LGLL) is characterized by clonal expansion of mature Natural Killer cells or T lymphocytes. All in all, the clinical presentation of LGLL is variable and heterogeneous. T-LGLL and NK-LGLL are clinically similar and both have chronic courses [8]. At the time of diagnosis, one-third of the patients are asymptomatic. Often, mild lymphocytosis in the blood count as an incidental finding, providing a reason for further diagnosis [6]. Presumably, symptoms might occur as result of different forms of cytopenia. Neutropenia occurs in 40–60% of cases and can lead to secondary or recurrent infections [5,9]. Furthermore, anemia with symptoms of weakness or transfusion indication may develop and splenomegaly occurs in 20–50% of cases [5].

Interestingly, LGL leukemia is characterized by a variety of concomitant diseases, of which autoimmune diseases are particularly common. Rheumatoid arthritis (RA) is the most frequently associated autoimmune disease [5,6,8]. Other autoimmune diseases include collagenosis, vasculitis, autoimmune cytopenia, and endocrinopathies [8]. However, the link between autoimmune phenomena and chronic proliferation of mature cytotoxic cells is only poorly understood. In the context of RA, in some cases, the diagnosis of T-LGLL might even be missed [41], and hence further testing should be considered in patients with RA and neutropenia. In addition, concomitant neoplasms also occur, most commonly B-cell neoplasms, such as non-Hodgkin lymphomas, multiple myeloma, or chronic lymphocytic leukemia, and associated myelodysplastic syndrome is also observed without knowledge of the causal connection [8]. Aggressive NK leukemia presents with systemic symptoms such as B symptoms, hepatosplenomegaly, lymphadenopathy, thrombocytopenia, and anemia [42].

T- and NK-LGLL are considered indolent diseases [6]. However, in the course of the disease, the majority of the patients require therapy [8]. Therapeutic indications include severe neutropenia, moderate neutropenia with recurrent infections, relevant anemias, or a strong increase in circulating LGL cells, as well as the manifestation of associated autoimmune phenomena requiring treatment [6,7]. Due to limited case numbers and a lack of prospective studies, LGLL therapy is based on empirical data. First-line therapy relies on the use of single immunosuppressive agents such as methotrexate (MTX) or cyclophosphamide. Switching between the two medications might be indicated in the absence of an apparent response. In addition, cyclosporine A (CyA) is used as a reserve drug if patients do not respond to MTX and cyclophosphamide [5]. Based on retrospective studies, the overall response rate appears quite broad, ranging from 21% to 85%, with similar responses to each of the three drugs [6]. Thus, therapy is individually adapted based on the prevailing symptoms and the side-effect profile of the drugs used [5].

Steroid therapy can be used as an adjunct and might lead to rapid normalization of blood counts [5]. Limited data are available for second-line therapy to treat patients who are refractory to the first-line agents. The benefit of purine analogs, combined chemotherapy, immunotherapy, splenectomy, or of targeted therapy addressing surface molecules, cytokines, or the Jak/Stat pathway is presently being addressed [6,7,43]. Nonetheless, there is an urgent need to develop new therapeutics specifically targeting LGL leukemia, because the disease remains incurable [6]. At present, the 10-year survival rate for chronic LGL leukemia is approximately 70% [8]. The most common cause of death is severe infection due to neutropenia. However, the prognosis of aggressive NK-LGL leukemia is very poor. It is refractory to most available therapeutic interventions and the median survival is only a few weeks [44].

As mentioned in the introduction, the classification of LGLL-subtypes is based on immunophenotype, cytology, and evidence of monoclonality. The most frequent T-LGLL phenotype is CD2^+^, CD3^+^, CD8^+^, CD4^−^, CD57^+^, CD45RA^+^, and CD16^+^ with or without expression of CD56, which is indicative of a constitutively active mature T-effector memory cell. αβ-TCRs are more frequent, but γδ-TCR^+^ cases have also been described. Only 10–15% of cases are CD4^+^ and CD8^−^ or CD8^+dim^. Notably, it was reported that based on histological inspection, most T-LGLL clones express cytotoxic effector proteins including granzymes and perforin, which presumably locate to LGLL-typical granules. Compared to T-LGLLs, NK-LGLLs seem to be more homogenous, are mostly categorized as CD2^+^, CD3^−^, CD8^+^, CD16^+^, CD56^+^, and also express cytotoxic effector molecules [6,7,8,9,10].

### 4.1. Expression and Storage of Cytotoxic Effector Proteins in LGLL

Although significant progress has been made in deciphering genetic and molecular alterations associated with LGLL, the contribution of LGLL-associated cytotoxicity and contributing effector molecules has only been incompletely addressed. In fact, it was reported that most if not all LGLL patients display constitutive expression of FasL gene transcripts and that leukemic cells from 90% of T-LGLL patients express both Fas (CD95) and FasL (CD178) at high levels, nonetheless being completely resistant to anti-Fas- and anti-CD3-mediated apoptosis. In addition, the high level of FasL was associated with pathology, most likely contributing to LGLL-associated neutropenia. Moreover, the increased serum level of sFasL in LGLL was proposed as a marker for disease activity [15,16,17,18], although sFasL is more likely involved in preventing Fas-mediated apoptosis [45].

Notably, in our analyses of four established LGLL cell lines, we did not detect FasL surface expression on any of the cell lines investigated, nor did we identify FasL in extracellular vesicles of MOTN-1 or NKL. However, Fas was readily detected in EV preparations (P25455, tumor necrosis factor receptor superfamily member 6), indicating its expression on LGLL cells and its release in extracellular vesicles. Notably, Fas abundance was significantly higher in EVs from MOTN-1. From our extensive studies on FasL in untransformed or leukemic cells, we know that due to its complex storage and release regulation and its high efficacy as a membrane-bound death factor [46], it seems rather unlikely that a leukemic population constitutively expresses high levels of unmutated functionally competent versions of this molecule. The reported presence of elevated levels of sFasL in LGLL patients’ sera might nevertheless indicate a substantial production and release of cytotoxic granules from leukemic cells. Although sFasL has been correlated with the prevention of apoptosis, in leukemic cells, the exposure or release of vesicles carrying the death ligand as a transmembrane molecule might have broader implications, including cell death induction. Notably, unsafe exposure of molecules transported in secretory vesicles or a release of LREVs as extracellular vesicles might contribute to immune dysfunction and to the development of autoimmune phenomena which have been associated with LGLL. In addition, soluble cytotoxic effector molecules might also contribute to symptoms of LGLL. For example, cytopenia might be caused by a release and infiltration of soluble cytotoxic effector molecules and/or cytokines into the bone marrow, since the degree of marrow infiltration with leukemic cells does not correlate with the degree of cytopenia in peripheral blood [6].

Granzyme B and perforin are often mentioned as additional markers to ascertain LGLL diagnosis, e.g., in histological staining of bone marrow biopsies [5]. For granulysin, another key effector of T and NK cells, the distribution, release, or impact in LGLL has not been investigated so far. We therefore addressed the distribution and release of LREV-associated cytotoxic effector molecules in established LGLL cell lines. Previously, we compared intracellular effector vesicles from the patient-derived NK LGLL line NKL with those from the NK cell lymphoblastic leukemia/lymphoma line YT and from untransformed cells. We reported heterogeneous profiles of functionally relevant proteins (including cytotoxic effector proteins) in individual leukemic and activated human NK cell populations [22]. In the present study, we followed this route and analyzed patient-derived LGLL cell lines with a primary focus on cytotoxic effector molecules. We chose the well-characterized T-LGLL MOTN-1 [23], the NK-LGLL lines NKL [19] and NK-92 [24], and the ANKL KHYG-1 [25] for our comparative studies. We analyzed the distribution of other lysosome-associated effector proteins by conventional and imaging flow cytometry or Western blotting. In contrast with FasL, high amounts of dipeptidyl peptidase 4 (DPP4, CD26) were detected on all tested LGLL cells. Notably, we recently reported that CD26 is not only a transmembrane signal transducer but is also associated with LREVs and released by activation-induced degranulation [31]. Not surprisingly, in the present study, we also found DPP4 in EVs of both MOTN-1 and NKL cells (P27487, dipeptidyl peptidase 4), highlighting that DPP4 is released as an active enzyme with extracellular vesicles from LGLL cells. Further studies might address the role of this dipeptidase in disease development and pathology [47].

To address the presence of cytotoxic effector molecules in LGLL-derived cell lines, we initially performed intracellular staining for granzymes (GrzA and GrzB) and Perforin (Prf). The two NK-LGLL lines NK-92 and NKL were positive for both granzymes and perforin, with a slightly higher fluorescence intensity for GrzA in NKL. In KHYG-1, we detected GrzA and Prf, but not GrzB. As expected, MOTN-1, a T-LGLL presenting no cytotoxic activity [23], did not stain for any of the tested effector proteins.

In previous analyses, the premature 15 kDa and the mature 9 kDa species of Gnly served as markers for distinct LREV entities [28]. We first enriched LREVs by subcellular fractionation and stained the resulting Western blots for the presence of granulysin, granzyme B, and LAMP-1. These analyses already pointed to a differential distribution of LREVs carrying individual effector molecules. In all cells, we detected LAMP-1 as a marker for lysosomes in the complete lysosomal fraction and in most individual subcellular fractions with occasional qualitative differences. The distribution of GrzB and Gnly presented rather heterogeneously. GrzB was present in the crude lysosomal fractions of both NK-LGLL and the ANKL cell line. However, whereas in NK-92, GrzB was enriched in heavy fractions (i.e., fraction 6 vesicles), it was much less intense in KHYG-1 and hardly detectable in NKL LREVs. As expected, GrzB was not detected in LREVs from MOTN-1.

Notably, both Gnly species were stained in the crude lysosomal fraction of all four cell lines. The distribution observed in NK-92 corresponded to what we described for untransformed T cells [28]. The mature 9 kDa Gnly was primarily detected in heavier fractions associated with GrzB, whereas 15 kDa Gnly was more abundant in the lighter fractions 1 to 3. Interestingly, in NKL, KHYG-1 and MOTN-1, the mature Gnly was associated with the lighter fractions 1 to 4 and in KHYG-1 and NKL, although present in distinct fractions, both 9 and 15 kDa Gnly were associated with GrzB. Given the reported high similarity in protein distribution in LREVs from untransformed cells, these results indicate that subcellular protein sorting and distribution of effector proteins might be altered in transformed LGLL cells. These apparent differences are also reflected by the analyses of intracellular protein storage via imaging flow cytometry. It is well established that in untransformed cytotoxic lymphocytes effector proteins including granzymes, perforin and 9 kDa granulysin are safely stored in intracellular secretory granules where they colocalize with the lysosomal marker protein LAMP-1 [27,28,40]. Interestingly, GrzB hardly colocalizes with LAMP-1 in both NKL and NK-92 cells. Moreover, in NK-92 cells, 9 kDa is hardly detectable in LAMP-1-positive intracellular granula, whereas in contrast with untransformed cells, 15 kDa Gnly is stored in intracellular lysosomal structures in a substantial number of cells. Although the overall Gnly expression is much lower in NKL cells, Gnly^+^ cells seem to store both 15 and 9 kDa in secretory granules. Thus, compared to untransformed cytotoxic lymphocytes, both NKL and NK-92 cells might display an altered effector protein sorting or storage machinery.

### 4.2. Differential Activation-Induced Release of 9 or 15 kDa Granulysin

We reported before that in untransformed T cells, individual LREVs containing either 15 kDa or 9 kDa Gnly utilize either Ca^2+^-independent non-classical or Ca^2+^-dependent classical degranulation for their mobilization and release [40]. In view of the altered LREV storage patterns in LGLL cells, we investigated signal requirements for the release of the two Gnly forms from NKL and NK-92. We analyzed the release into culture supernatants by immunoprecipitation and Western blotting after stimulation with TPA to trigger non-classical or TPA/ionomycin to trigger classical degranulation. Immunoprecipitates with the polyclonal anti-Gnly antibody from cell lysates of NKL and NK-92 revealed that the total levels of both forms of intracellular Gnly might be comparable. Interestingly, we found that NKL cells hardly release any Gnly upon stimulation with TPA and/or ionomycin. In contrast, TPA activation resulted in a selective release of the 15 kDa variant from NK-92 cells, whereas TPA and ionomycin induced the release of both Gnly forms. Importantly, this Ca^2+^-dependent release of 9 kDa Gnly triggered by TPA/ionomycin was reduced by Ca^2+^ chelation with EGTA. These results again highlight the more heterogenous and altered storage and release patterns of cytotoxic effector proteins in LGLL cells.

### 4.3. Proteomic Profiling of LGLL-Derived Extracellular Vesicles

The MS-based proteomic characterization of EV samples is generally challenging, due to the high number of expected integral membrane proteins and the limited sample amounts. While the depth of analysis, i.e., the number of protein identifications is dependent on multiple factors, such as the cell type, the EV release or the enrichment method, the presented dataset with 1742 identified and 1174 quantified proteins provides a comparable size to other publications in the field [48,49]. Notably, for our comparative analysis of T- and NK-LGLL-derived extracellular vesicles, we collected constitutively released particles from MOTN-1 and NKL cells over a culture period of 72 h in exosome-reduced medium. The isolated EVs presented as homogeneous populations of small- to medium-size vesicles of 170–200 nm in diameter in NTA analyses. Interestingly, both cell types released comparable amounts of vesicles of similar size, resulting in comparable sample material for the subsequent MS analyses.

The statistical testing revealed that more than half of the identified proteins (660) provided no significant abundance difference in the EV preparations of MOTN-1 and NKL, while 186 proteins (119 strict and 67 moderate) presented significantly higher abundance levels in MOTN-1-derived EV and 263 proteins (173 strict and 90 moderate) higher levels in NKL-derived EV. Moreover, 44 proteins were exclusively identified in MOTN-1 EV and 21 proteins exclusively in NKL-derived vesicles (see Supplementary Table S2 for individual protein annotations).

A total of 766 (43.9%) of the identified proteins were associated with the GO term “extracellular exosomes” (GO:0070062). These included common marker proteins forming the core proteome of small extracellular vesicles/exosomes such as the proteins involved in exosome biogenesis including TSG101 or the programmed cell death 6-interacting protein/Alix and syntenin-1, which was recently proposed as a putative universal biomarker for exosomes [49]. Notably, in line with our previous analyses of T or NK cell-derived exosomes, we identified the tetraspanins CD63 and CD81, but not CD9 in EVs from MOTN-1 or NKL. However, several Rab GTPases, which are commonly associated with lysosomal trafficking and EV formation (i.e., Rab5B, Rab7A, Rab11B and Rab27A) and the lysosome-associated membrane proteins LAMP-1 (CD107a) and LAMP-2 (CD107b), were found in both EV populations. Here, LAMP-1 showed increased abundance levels in MOTN-1 samples, whereas no significant difference was observed for LAMP2. Other characteristic and commonly EV-associated proteins such as flotillins, annexins, heat shock proteins, cell adhesion molecules or integrins (i.e., integrin β1), CD26/DPP4, CD47, and ADAM proteases were also detected in comparable abundances in EVs from both NKL and MOTN-1. For these protein families, a frequent association or enrichment in exosomes from different cellular sources has been documented [49].

Unexpectedly, 2B4 (CD244 [50]) and 2DL4 (CD158d [51]), two molecules which have been described as activating or inhibiting NK cell receptors, were identified in the EVs of both NKL and MOTN-1 without significant differences in abundance, while the inhibitory receptor LILRB1 [52] was detected with a higher abundance level in NKL-derived vesicles.

Importantly, proteins with higher abundance levels in NKL-derived EVs also included the mentioned cytotoxic effector proteins GrzA, GrzB and Prf. This is in line with our own analyses and corresponds to the cytotoxic potential of this NK-LGLL cell line. In contrast, MOTN-1-derived EVs presented the T-cell surface glycoprotein CD4 as an indicator for the original T-cell lineage and the TNF-receptor 2 in higher abundance. Also, the death receptor Fas (CD95) was detected in EVs of both cell lines; however, its presence significantly increased in the MOTN-1-derived vesicles. Interestingly, GrzK was exclusively identified in EV of MOTN-1. This granzyme is usually present in granules of NK cells and cytotoxic T lymphocytes and supposedly operates as a cytotoxic pro-apoptotic serine protease towards foreign, infected, or malignant cells. However, numerous other intracellular and extracellular roles for GrzK have been identified since its initial cloning as granzyme 3 in 1995 [53,54]. GrzK is the only other tryptase within the granzyme family besides GrzA and has long been regarded as a redundant replacement for GrzA. Notably, GrzK is activated by removing a signal dipeptide which directs the pre-pro-GrzK to the endoplasmic reticulum (ER). Upon activation by granular cathepsin (or dipeptidyl-peptidases?), GrzK cleaves diverse substrates that were also identified in EVs from LGLL cells, including nucleosome assembly protein, heterogeneous nuclear ribonucleoprotein (hnRNP) K, β-tubulin, and α-tubulin. Similar to the other granzymes, however, the “traditional” role of GrzK is debated and extracellular functions of GrzK in promoting inflammation and infections are emerging (see [54] for a review). As mentioned, GrzK was exclusively identified in MOTN-1 vesicles, which in turn contained much less GrzA, GrzB, and Prf compared to NKL-derived EV. In essence, MOTN-1 represents a CD4^+^ T-LGLL that does not display significant cytotoxicity while carrying an atypical set of granular effector molecules.

Along this line, when GO terms were linked to each individual protein, MOTN-1 EVs were significantly enriched for GO terms such as protein folding and antigen presentation. Interestingly, proteins associated with membranes (external side of plasma membrane, intrinsic to endoplasmic reticulum membrane, integral to organelle membrane) were also increased in MOTN-1. In contrast, in EVs of NKL, the GO terms protein–DNA complex and nucleosome were enriched, pointing to DNA-interacting proteins such as histones. Moreover, terms attributed to the cytoskeleton and to cell motility were enriched in NKL samples.

In conclusion, as previously reported for intracellular LREVs from different NK cell lines and activated NK cells [22], our data collection points to a clearly clonotypic distribution of distinct effector molecules in individual LGLL cell lines.

## 5. Conclusions

Large granular lymphocyte leukemias (LGLL) originate from mature CD3^+^ T effector memory cells or CD3^−^ NK cells and are characterized by containing large granules that store and transport cytotoxic effector molecules inter alia. LGLL cells frequently display uncontrolled proliferation and cytotoxicity, both resulting in chronic malignancy and autoimmunity. We demonstrated that individual patient-derived cell lines of different LGLL-subtypes carry or release individual and clonotypic combinations of effector molecules including, e.g., granzymes, perforin, and granulysin. Importantly, the individual distribution of effectors was also detected in proteome profiles of constitutively released extracellular vesicles (EVs) of two representative LGLL cell lines. We conclude that LGLL-derived EVs might be regarded as valuable targets for the analysis of proteins that contribute to LGLL pathology in individual patients and thus might open new routes for individualized therapeutic interventions.

## Figures and Tables

**Table 1:** Proteins in EV of NKL and MOTN-1. Short list of characteristic proteins that were identified in EV of the LGLL cell lines NKL and MOTN-1 (for complete list, see Supplementary Table S1).

Accession	Name	Gene
P04083	Annexin A1	*ANXA1*
P07355	Annexin A2	*ANXA2*
P50995	Annexin A11	*ANXA11*
O75955	Flotillin-1	*FLOT1*
Q14254	Flotillin-2	*FLOT2*
P07900	Heat shock protein HSP 90-alpha	*HSP90AA1*
P34932	Heat shock 70 kDa protein	*HSPA4*
P61020	Ras-related protein Rab-5B	*RAB5B*
P51149	Ras-related protein Rab-7A	*RAB7A*
Q15907	Ras-related protein Rab-11B	*RAB11B*
P51159	Ras-related protein Rab-27A	*RAB27A*
Q9H4M9	EH domain-containing protein 1	*EHD1*
Q9H223	EH domain-containing protein 4	*EHD4*
O14672	Disintegrin and metalloproteinase domain-containing protein 10	*ADAM10*
P78325	Disintegrin and metalloproteinase domain-containing protein 8	*ADAM8*
P08962	CD63 antigen	*CD63*
P60033	CD81 antigen	*CD81*
Q8WUM4	Programmed cell death 6-interacting protein	*PDCD6IP*
Q99816	Tumor susceptibility gene 101 protein	*TSG101*
O00560	Syntenin-1	*SDCBP*
P05362	Intercellular adhesion molecule 1	*ICAM1*
P19320	Vascular cell adhesion protein 1	*VCAM1*
P13612	Integrin alpha-4	*ITGA4*
P05556	Integrin beta-1	*ITGB1*
P05107	Integrin beta-2	*ITGB2*
Q15762	CD226 antigen	*CD226*
P11279	Lysosome-associated membrane glycoprotein 1	*LAMP1*
P13473	Lysosome-associated membrane glycoprotein 2	*LAMP2*
P27487	Dipeptidyl peptidase 4	*DPP4*
Q9BZW8	Natural killer cell receptor 2B4	*CD244*
Q99706	Killer cell immunoglobulin-like receptor 2DL4	*KIR2DL4*
Q8NHL6	Leukocyte immunoglobulin-like receptor subfamily B member 1	*LILRB1*
P25445	Tumor necrosis factor receptor superfamily member 6	*FAS*

**Table 2:** Proteins with significant abundance differences in EVs of NKL and MOTN-1. Top ten proteins that were either enriched in EVs of NKL or in EVs of MOTN-1. Negative log_2_(FC) values indicate a higher protein abundance in NKL; positive values refer to higher abundance in MOTN-1 samples.

Accession	Name	Gene	log_2_(FC)
	Higher Protein Abundance in NKL		
P04229	HLA class II histocompatibility antigen, DRB1-1 beta	*HLA-DRB1*	−8.99
P10144	Granzyme B	*GZMB*	−6.44
Q15942	Zyxin	*ZYX*	−6.23
P05161	Ubiquitin-like protein ISG15	*ISG15*	−5.42
Q9H4G4	Golgi-associated plant pathogenesis-related protein 1	*GLIPR2*	−4.82
Q12792	Twinfilin-1	*TWF1*	−4.51
P32246	C-C chemokine receptor type 1	*CCR1*	−4.46
Q9NPY3	Complement component C1q receptor	*CD93*	−4.42
Q8IUE6	Histone H2A type 2-B	*HIST2H2AB*	−4.40
Q8NHL6	Leukocyte Ig-like receptor subfamily B member 1	*LILRB1*	−4.36
	**Higher protein abundance in MOTN-1**		
P31785	Cytokine receptor common subunit gamma	*IL2RG*	4.33
P20333	Tumor necrosis factor receptor superfamily member 1B	*TNFRSF1B*	4.74
P49961	Ectonucleoside triphosphate diphosphohydrolase 1	*ENTPD1*	4.87
Q96C23	Aldose 1-epimerase	*GALM*	5.05
P13762	HLA class II histocompatibility antigen, DR beta 4 chain	*HLA-DRB4*	5.20
P13760	HLA class II histocompatibility antigen, DRB1-4 beta chain	*HLA-DRB1*	6.48
O15031	Plexin-B2	*PLXNB2*	6.70
P01730	T-cell surface glycoprotein CD4	*CD4*	6.76
P32119	Peroxiredoxin-2	*PRDX2*	7.43
Q30134	HLA class II histocompatibility antigen, DRB1-8 beta	*HLA-DRB1*	7.45

## Data Availability

All proteomics raw data have been uploaded to the ProteomeXchange Consortium [38] via the PRIDE partner repository with the dataset identifier PXD053228.

## Supplementary Materials

The following supporting information can be downloaded at https://www.mdpi.com/article/10.3390/cells13161310/s1, Supplementary_Proteomics_Tables, containing Supplementary Table S1—List of high-confidence protein identifications; Supplementary Table S2—Results of statistical Welch *t*-test with Benjamini-Hochberg FDR, q = 0.01; Supplementary Table S3—Results of Fisher’s exact test with Benjamini-Hochberg FDR, q = 0.05; Supplementary Table S4—Results of 1D annotation enrichment analysis with Benjamini-Hochberg FDR, q = 0.05; Supplementary Table S5—Results of Fisher’s exact test with Benjamini-Hochberg FDR, q = 0.05 (selected EV proteins).
